# Changes in multimorbidity burden and their impact on patient and healthcare outcomes in people with HIV over a 3–5-year period

**DOI:** 10.1097/QAD.0000000000004260

**Published:** 2025-06-04

**Authors:** Luxsena Sukumaran, Alan Winston, Frank A. Post, Jane Anderson, Marta Boffito, Memory Sachikonye, Patrick W.G. Mallon, Laura Waters, Jaime Vera, Fiona Burns, Caroline A. Sabin

**Affiliations:** a Institute for Global Health, University College London; b National Institute for Health and Care Research (NIHR) Health Protection Research Unit (HPRU) in Blood-borne and Sexually Transmitted Infections at University College London; c Department of Infectious Disease, Imperial College London; d King's College Hospital NHS Foundation Trust; e Homerton University Hospital; f Chelsea and Westminster Healthcare NHS Foundation Trust; g UK Community Advisory Board (UK-CAB), London, UK; h University College Dublin, Dublin, Ireland; i Mortimer Market Centre, Central and Northwest London NHS Foundation Trust, London; j Brighton and Sussex Medical School, Brighton; k Royal Free London NHS Foundation Trust, UK.

**Keywords:** comorbidity, health outcomes, HIV, multimorbidity, multimorbidity patterns, principal component analysis

## Abstract

**Background:**

Despite increasing multimorbidity among people with HIV, its impact on health outcomes over time remains uncertain. We explored how distinct multimorbidity patterns affect a broad range of health outcomes over a 3–5-year period.

**Methods:**

Principal component analysis (PCA) was used to identify multimorbidity patterns at baseline. Burden *z*-scores were calculated for each individual/pattern at baseline and a follow-up visit, and the differences in scores over time were examined. Participants completed health assessments including questionnaires [physical/mental health (SF-36)], depressive symptoms (CES-D and PHQ-9, falls, frailty and healthcare utilization), cognitive testing and pain mannequins tests. Multivariable regression models assessed associations between changes in morbidity burden *z*-scores and health outcomes.

**Results:**

Six multimorbidity patterns were identified in 1073 participants: “*cardiovascular disease” (CVD)*, “*sexually transmitted infections” (STIs)*, “*metabolic”*, “*mental/joint”*, “*neurological”*, and “*cancer/other”*. Subsequent analyses included 793 participants (median [interquartile range; IQR] age 53 [47–59] years; 86% male; 97% on ART) with follow up data. *CVD* and *metabolic* burden were associated with specialist appointments (CVD: β = 1.47; metabolic: β = 1.53, *P* < 0.01) and ED visits (CVD: β = 1.44; metabolic: β = 1.89, *P* < 0.01), *mental/Joint* and *neurological* burden with poorer physical and mental health, frailty and recurrent falls (*P* < 0.01), and *cancer/other* burden with higher depressive scores (β = 3.28, *P* < 0.001), widespread pain (odds ratio, OR = 2.20, *P* < 0.001), and hospital visits (OR = 2.31, *P* < 0.001).

**Conclusion:**

Distinct morbidity patterns differentially affected health outcomes and healthcare utilization over time, underscoring the need for targeted, integrated care to improve quality of life and address their complex needs.

## Introduction

In many settings, the scale-up of antiretroviral therapy (ART) has markedly improved the life expectancy of individuals with HIV [1]. However, as people with HIV age, the prevalence of multiple long-term conditions (MLTCs), or multimorbidity, is rising. A modelling study in the Netherlands estimated that the proportion of people with HIV living with three or more noncommunicable diseases will rise from 0.3% in 2010 to 28% by 2030 [2]. MLTCs add complexity to HIV management and carry significant implications, including reduced quality-of-life and increased utilization of healthcare services [3–6]. These challenges are compounded by healthcare systems and guidelines that primarily focus on individual conditions [7], leading to a fragmented, “disease-oriented” approach that can result in poorly coordinated care.

Evidence on the impact of MLTCs on health outcomes among people with HIV is limited, with most studies focusing on individual conditions or disease categories, such as cardiovascular disease (CVD). However, increasingly, studies demonstrate that certain conditions frequently co-occur in distinct patterns, driven by shared risk factors or pathophysiological pathways [8]. Understanding these patterns and their implications for health outcomes is crucial for developing effective, integrated care strategies. Our prior work using the Pharmacokinetic and clinical Observations in PeoPle over fiftY (POPPY) cohort underscored the importance of investigating multimorbidity patterns, showing that certain combinations of comorbidities, such as those involving CVDs and mental health problems, were associated with poorer physical health, functional impairment and hospitalization [9]. However, the analysis was limited to a narrow set of health outcomes (patient-reported physical and mental health, number of general practitioner (GP) visits, hospitalization and functional impairment), limiting our understanding of the broader health impacts of multimorbidity, including frailty, cognitive problems and chronic pain in this population. Broadening the scope to include a wider range of outcomes can provide a more comprehensive understanding for developing effective targeted care strategies for the unique and evolving healthcare needs of this population. Furthermore, to our knowledge, the longitudinal effects of multimorbidity on health trajectories and healthcare demands remain unexplored. Understanding these long-term effects can reveal how co-occurring conditions impact health deterioration and healthcare utilization in ways that may differ from single-disease trajectories.

To address these gaps, the present study investigated associations between multimorbidity patterns and a broad range of patient-related and healthcare outcomes over a 3–5 year follow-up period among POPPY participants with HIV.

## Methods

### Study participants and procedures

The POPPY study is a prospective cohort study of individuals living with and without HIV from the United Kingdom and Ireland. Information on participant characteristics and cohort eligibility criteria is described elsewhere [10]. Briefly, the study enrolled three groups of participants between April 2013 and January 2016: older participants with HIV (aged *≥*50 years), younger participants with HIV (18–49 years), and HIV-negative controls (aged *≥*50 years, frequency-matched to the older people with HIV based on gender, ethnicity, sexual orientation, and location). The present analysis focused only on participants with HIV. During two study visits (baseline and wave 3, conducted 3–5 years later), data on socio-demographic factors, comorbidities, and laboratory measurements were collected by trained clinical staff and through data linkage with the UK Collaborative HIV Cohort (UK CHIC) study and the Mater Misericordiae University Hospital (MMUH) Infectious Diseases (ID) Cohort in Dublin.

### Data on comorbidities

Participants provided information on comorbidities during structured interviews. Whenever possible, self-reported information was cross-checked with data on concomitant medications and healthcare utilization (e.g., visits to general practitioners, hospitals and specialists). Participants were asked about 52 specific health conditions and were able to mention any other conditions they experienced through free-text questions. The present analysis includes 70 comorbidities selected based on prevalence (≥1.5% in the study population) and clinical relevance to people with HIV (see Supplementary Table 1, Supplemental Digital Content).

### Health outcomes

All POPPY participants completed a range of questionnaires during each study visit. The Short Form Health Survey (SF-36) questionnaire was used to obtain physical and mental health summary scores [11]. Briefly, scores from eight subscales were standardized into *z*-scores (mean = 0, standard deviation = 1) using sex-specific and age-specific means and standard deviations from the 1996 Health Survey for England dataset [12], then combined to obtain a physical and mental health summary score, rescaled with a mean of 50 and a standard deviation of 10. Frailty was assessed using a modified version of Fried's criteria [13,14], that includes four of the five components of the original score (grip strength, time taken to walk 15 feet, self-reported exhaustion, and self-reported low physical activity). Individuals who met ≥3 of these criteria were defined as frail. Recent (past 28 day) fall history was elicited through the Cambridge Falls questionnaire [15], with individuals reporting ≥*2* falls over this period considered to have experienced a recurrent fall. Functional impairment was defined as impairment in ≥1 daily activities using the Lawton Instrumental Activities of Daily Living (IADL) questionnaire [16]. Cognitive function was assessed using the CogState battery, covering six cognitive domains: visual learning, psychomotor function, visual attention, executive function, verbal learning and working memory. Briefly, a global T-score was obtained by averaging all domain T-scores for an individual; a higher T-score indicates better cognitive function. Current/severity of depressive symptoms were measured using CES-D and PHQ-9 questionnaires [17,18]. Using standard cut-offs, participants with PHQ-9 scores were categorized as having no (≤4), mild (5–9), moderate (10–14) or severe (≥15) depressive symptoms [18]. Self-reported pain and a pain mannequin identified widespread pain following 2019 American College of Rheumatology fibromyalgia criteria [19], requiring pain in at least four of the five regions [(1) left and (2) right shoulder/upper arm/lower arm, (3) left and (4) right hip/upper leg/lower leg and (5) neck/upper back/lower back] and in at least seven of 15 body sites. Medication use was documented during the baseline study visit. Healthcare utilization, including hospital visits (procedures such as cancer treatments, injections, surgeries, transfusions, or investigations like blood/urine tests, ultrasounds, X-rays, physiotherapy or screenings) and counts of GP, hospital specialist, and Emergency Department (ED) visits in the preceding year, was also assessed at both study visits.

### Statistical analysis

As described previously [20,21], we used principal component analysis (PCA) to identify patterns of multimorbidity. Based on recommendations to include a broader range of clinically relevant conditions and those commonly associated with multimorbidity in this population [22], we expanded the number of comorbidities considered from 65 to 70, which resulted in some changes in the composition of the identified patterns [21] compared to earlier work [20]. Pairwise associations between 70 comorbidities reported at baseline were calculated using Somers’ *D* statistic [23], and PCA was applied to this matrix to extract principal components (PCs) that represent nonrandom groups of commonly co-occurring comorbidities. An *oblimin* rotation was used to allow patterns to be associated with each other and thus, multiple patterns to be present within the same individual. The optimal number of PCs or patterns was determined through scree plots and the very simple structure (VSS) criterion. We evaluated a range of correlation/loading thresholds (0.10–0.40) and selected ≥0.25 as a threshold to determine comorbidities that were significantly associated with each pattern, guided by clinical relevance (not shown).

To measure multimorbidity burden over time, we calculated morbidity burden z-scores for each participant/pattern at baseline and visit 3, based on data on the presence/absence of comorbidities with a correlation greater than 0.25 for each pattern. Importantly, participants were not assigned to exclusive groups; rather, each individual could have a score for multiple patterns depending on their reported comorbidities. Both visit scores were standardized using the baseline mean and standard deviation (SD), with a 1-unit change representing a 1 SD increase in the morbidity burden relative to the baseline mean, ensuring consistent interpretation across all patterns. Burden *z*-scores above 0 indicate a higher morbidity burden than the sample mean. The change in burden for each pattern/participant was calculated as the difference between the z-scores from both visits. This was calculated for all patterns except the *STIs* pattern, which was excluded from longitudinal analysis because STIs are generally acute conditions that are not expected to persist or reflect a chronic disease burden over time.

The associations between changes in morbidity burden *z*-scores for each pattern and each health outcome was analysed using different regression models. Linear regression was used to investigate the relationship of burden *z*-scores with four continuous outcomes: SF36 physical summary scores; SF36 mental health summary scores; cognitive global T-scores; CES-D scores. Regression coefficients represent an average increase/decrease in the outcome associated with a 1-SD increase in the burden score of a pattern. Logistic regression, with ORs reported, was used for the following outcomes: frailty, recurrent falls, functional impairment, widespread pain and hospital visits. PHQ-9 scores were analysed using ordinal logistic regression, with ORs reported. Negative binomial regression was used for the number of GP, ED and hospital specialist visits, and IRRs associated with a 1-SD increase in burden scores are reported. This model was chosen over Poisson regression to account for the overdispersion observed in the count outcome variables.

The present analysis focused on participants with complete data on relevant covariates (age, gender, race, smoking status, alcohol status, recreational drug use, nadir CD4^+^ cell count and years since HIV diagnosis) and health outcomes at the wave 3 visit. For all models, univariable associations were examined first (Level 1). Next, *a priori* selected demographic and lifestyle factors (age at baseline, gender, ethnicity, smoking status, alcohol use and recreational drug use in the last six months) were included (Level 2). *A priori* selected HIV-related variables (nadir CD4^+^ T-cell count and years since HIV diagnosis) were also explored in the multivariable models (included in the supplementary material). *P*-values <0.05 were considered statistically significant.

The analyses were performed using R V4.2.4 (R Foundation for Statistical Computing, Vienna, Austria).

## Results

### Characteristics of study participants

Among the 1073 participants with HIV enrolled in POPPY at baseline, 834 (78%) attended a wave 3 visit. Of these, 793 (95%) provided complete data on the relevant covariates. The sociodemographic, lifestyle and HIV-related characteristics of included participants are summarised in Table 1. Participants were predominantly male (85.6%), of white ethnicity (85.5%), men who have sex with men (MSM; 77.2%) with a median (IQR) age of 53 (47, 59) years. Most participants were on ART (97.1%) with an undetectable viral load (<50 copies/ml; 92.2%). The median (IQR) nadir CD4^+^ T-cell count and years since HIV diagnosis were 205 (108–310) cells/mm^3^ and 13.2 (7.7–20.4) years, respectively. The median (IQR) time between the POPPY enrollment (baseline visit) and follow-up visit was 26 (24–30) months (range: 16–54 months).

**Table 1:** Baseline socio-demographic, lifestyle and HIV-related characteristics of included POPPY participants with HIV.

Characteristic *n* (%) or median (IQR)	Total (*n* = 793)
Age (years)	53 (47–59)
Gender	
Male	679 (85.6)
Female	114 (14.4)
Ethnicity	
Black-African	115 (14.5)
White	678 (85.5)
Sexual orientation	
MSM	612 (77.2)
Heterosexual	181 (22.8)
BMI (kg/m^2^)	25.5 (23.2–28.2)
Smoking status	
Never	330 (41.6)
Past	275 (34.7)
Current	188 (23.7)
Alcohol use	
Never	64 (8.1)
Past	91 (11.5)
Current	638 (80.5)
HIV-related factors	
Undetectable viral load (HIV RNA < 50 copies/mL)	725 (91.4)
Years since HIV diagnosis	13.2 (7.7–20.4)
On ART	774 (97.6)
Nadir CD4^+^ cell count (cells/mm^3^)	205 (108–310)

ART, antiretroviral therapy; BMI, body mass index; IQR, interquartile range; MSM, men who have sex with men; RNA, ribonucleic acid.

### Multimorbidity patterns and burden *z*-scores

Using PCA and baseline data, six multimorbidity patterns were identified, accounting for 23.3% of the total variance in the 70 comorbidities: *CVDs*; *STIs*; *metabolic*; *mental/joint*; *neurological*; and *cancer/other*. The comorbidities strongly correlated with each pattern are reported in Supplementary Table 2, Supplemental Digital Content.

**Fig. 1:**
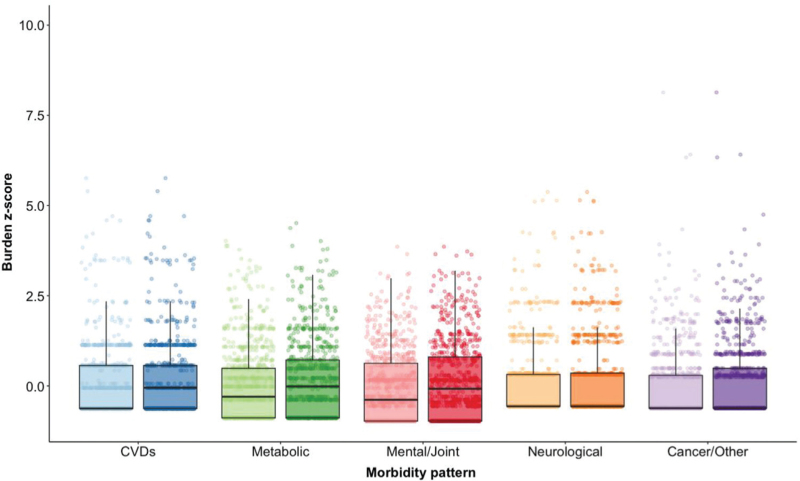
Distribution of baseline (lighter colour) and wave 3 (darker colour) morbidity burden *z*-scores among all POPPY participants with HIV who returned for the wave 3 visit (*n* = 793).

Morbidity burden z-scores were then generated for each participant and each pattern at baseline and follow-up (excluding the *STIs* pattern) visits. Median (IQR) burden *z*-scores at baseline (in decreasing order) were −0.30 (−0.89, 0.49), −0.38 (−0.97, 0.63), −0.56 (−0.56, 0.32), −0.62 (−0.62, 0.56), −0.62 (−0.62, 0.29) for *metabolic, mental/joint, neurological, CVDs* and *cancer/other*, respectively (Fig. 1). Median (IQR) burden *z*-scores at follow-up were −0.02 (−0.89, 0.72), −0.08 (−0.97, 0.81), −0.56 (−0.56, 0.35), −0.05 (−0.62, 0.56), and −0.62 (−0.62, 0.49) for these *z*-scores, respectively (Fig. 1).

### Health outcomes at visit 3

At visit 3, the median [IQR] physical and mental health score, cognitive global T-score and CES-D score were 52.7 [43.8–56.4], 51.4 [42.1–57.5], 48.2 [44.0–51.7] and 9 [3–21], respectively (Table 2). Median [IQR] counts for GP visits, hospital specialist and ED visits over the last 12 months were 2 [1–4], 2 [0–5] and 0 [0–1], respectively. Frailty, recurrent falls and functional impairment were observed in 23.1%, 16.4% and 12.9% of participants, respectively. Moderate or severe depressive symptoms (PHQ-9) were reported by 24.2% of participants. Widespread pain was reported by 13.4% of participants, and 67.7% had been hospitalized in the year preceding the study visit.

**Table 2:** Prevalence (*n*, %) or distribution (median [IQR]) of health outcomes considered at visit 3.

Health outcome *n* (%) or median (IQR)	Visit 3
SF-36 physical health summary score	52.8 (44.0–56.4)
SF-36 mental health summary score	51.4 (42.1–57.6)
Frailty	183 (23.1)
Recurrent falls	117 (16.4)
Functional impairment	98 (12.9)
Cognitive function	48.2 (44.0–51.7)
Depression	
CES-D score	9 (3–21)
PHQ-9	
None	381 (48.1)
Mild	145 (18.3)
Moderate	75 (9.5)
Severe	192 (24.2)
Widespread pain	88 (13.4)
Number of GP visits over the last 12 m	2 (1–4)
Number of specialist visits over the last 12 m	2 (0–5)
Number of ED visits over the last 12 m	0 (0–1)
Hospital visits in the preceding year	544 (65.2)

CES-D, Center for Epidemiologic Studies Depression Scale; ED, emergency department; GP, general practitioner; IQR, interquartile range; PHQ-9, Patient Health Questionnaire-9; SF-36, 36-Item Short Form Survey.

### Associations of changes in burden *z*-scores with health outcomes at wave 3

An increase of 1-unit in the *CVDs* burden *z*-score (indicating a 1 SD increase in CVD-related morbidity burden above the baseline mean) was associated with higher odds of functional impairment (OR [95% CI]: 2.00 [1.27, 3.14]), as well as a higher number of specialist (IRR [95% CI]: 1.47 [1.11, 1.94]) and ED (IRR [95% CI]: 1.44 [1.06, 1.94]) visits, and poorer physical health scores (β [95% CI]: −2.71 [−4.89, −0.52]), after adjustment for confounders (Tables 3 and 5). Similarly, a 1-SD increase in *metabolic* burden *z*-scores were linked to functional impairment (OR [95% CI]: 2.64 [1.45–4.81]) and an increased risk of hospital visits (OR [95% CI]: 2.86 [1.45–5.64]). Weaker associations were observed for frailty (*P* = 0.05). An increase in *mental/joint* scores was associated with higher odds of frailty (OR [95% CI]: 1.93 [1.27, 2.91]) and recurrent falls (OR [95% CI]: 1.72 [1.09, 2.74]), along with poorer physical and mental health scores (β [95% CI]: −3.91 [−6.05, −1.78] and β [95% CI]: −7.13 [−9.20, −5.07], respectively). These scores were also associated with higher CES-D scores (β [95% CI]: 7.08 [4.60–9.57]) and severe depression (OR [95% CI]: 1.93 [1.35, 2.76]) (Table 4). Increased *neurological* burden was associated with worse outcomes for most measures considered at wave 3, including higher odds of frailty, functional impairment and widespread pain, as well as poorer physical and mental health scores (all *P*'s < 0.001). Finally, increased *cancer/other* scores were associated with higher CES-D scores (β [95% CI]: 3.28 [1.28, 5.27]) and higher odds of widespread pain (OR [95% CI]: 2.20 [1.52, 3.19]) and hospital visits (OR [95% CI]: 2.33 [1.46, 3.71]) after adjustment.

**Table 3:** Longitudinal associations between changes in each morbidity burden z-scores and patient-related health outcomes from baseline to wave 3 (3–5 years later) in POPPY participants with HIV, assessed using linear or logistic regression models: Level 1 (unadjusted) and Level 2 (adjusted for age, sex, race, smoking, alcohol use and recreational drug use in the last six months).

	Patient-related health outcomes				
	
Change in burden *z*-scores	Physical health (*n* = 618) *Beta* coefficient (95% CI) *P*-value	Mental health (*n* = 618) *Beta* coefficient (95% CI) *P*-value	Frailty (*n* = 793) OR (95% CI) *P*-value	Recurrent falls (*n* = 714) OR (95% CI) *P*-value	Functional impairment (*n* = 758) OR (95% CI) *P*-value
*CVD*					
Level 1	**−2.43 (−4.62, −0.25) *P* = 0.03**	−1.35 (−3.52, 0.82) *P* = 0.22	1.25 (0.82, 1.90) *P* = 0.30	1.43 (0.89, 2.29) *P* = 0.14	**2.00 (1.27, 3.14) *P* *<* 0.001**
Level 2	**−2.71 (−4.89, −0.52) *P* = 0.02**	−1.38 (−3.56, 0.79) *P* = 0.21	1.16 (0.75, 1.78) *P* = 0.51	1.47 (0.91, 2.39) *P* = 0.12	**1.96 (1.22, 3.18) *P* = 0.01**
*Metabolic*					
Level 1	−1.17 (−3.94, 1.60) *P* = 0.41	**−2.96 (−5.70, −0.22) *P* = 0.03**	**1.92 (1.15, 3.20) *P* = 0.01**	0.98 (0.50, 1.92) *P* = 0.95	**2.75 (1.54,4.89) *P* *<* 0.001**
Level 2	−1.59 (−4.37, 1.20) *P* = 0.26	**−3.11 (−5.87, −0.36) *P* = 0.03**	1.69 (0.99, 2.89) *P* = 0.05	0.87 (0.44, 1.75) *P* = 0.71	**2.64 (1.45, 4.81) *P* *<* 0.001**
*Mental/joint*					
Level 1	**−4.24 (−6.38, −2.10) *P* *<* 0.001**	**−7.36 (−9.43, −5.30) *P* *<* 0.001**	**1.76 (1.19, 2.62) *P* = 0.01**	**1.78 (1.14, 2.77) *P* = 0.01**	**1.73 (1.04, 2.89) *P* = 0.04**
Level 2	**−3.91 (−6.05, −1.78) *P* *<* 0.001**	**−7.13 (−9.20, −5.07) *P* *<* 0.001**	**1.93 (1.27, 2.91) *P* *<* 0.001**	**1.72 (1.09, 2.74) *P* = 0.02**	**1.69 (1.00, 2.85) *P* = 0.05**
*Neurological*					
Level 1	**−4.52 (−6.11, −2.93) *P* *<* 0.001**	**−2.84 (−4.44, −1.24) *P* *<* 0.001**	**1.88 (1.41, 2.52) *P* *<* 0.001**	**1.47 (1.05, 2.06) *P* = 0.02**	**1.98 (1.43, 2.75) *P* *<* 0.001**
Level 2	**−4.55 (−6.13, −2.98) *P* *<* 0.001**	**−3.01 (−4.60, −1.43) *P* *<* 0.001**	**1.88 (1.39, 2.54) *P* *<* 0.001**	**1.49 (1.06, 2.10) *P* = 0.02**	**2.10 (1.50, 2.93) *P* *<* 0.001**
*Cancer/other*					
Level 1	**−2.75 (−4.44, −1.07) *P* *<* 0.001**	**−2.21 (−3.89, −0.54) *P* = 0.01**	**1.55 (1.14, 2.12) *P* = 0.01**	1.21 (0.84, 1.76) *P* = 0.31	**1.52 (1.05, 2.21) *P* = 0.03**
Level 2	**−3.07 (−4.74, −1.40) *P* *<* 0.001**	**−2.41 (−4.07, −0.75) *P* *<* 0.001**	**1.65 (1.20, 2.28) *P* *<* 0.001**	1.25 (0.86, 1.82) *P* = 0.25	**1.57 (1.07, 2.29) *P* = 0.02**

CI, confidence interval; CVDs, cardiovascular diseases; OR, odds ratio. A 1-unit increase in morbidity z-scores represents an increase in burden by 1 standard deviation above the baseline mean. Bold text indicates statistically significant associations (*P* < 0.05). A grayscale shading system is used to reflect the relative strength of associations: darker cells represent stronger effect sizes (higher beta coefficients or odds ratios), and lighter cells indicate weaker but still statistically significant associations. Unshaded cells represent nonsignificant associations. This visual distinction is intended to aid interpretability and highlight key findings.

**Table 4:** Longitudinal associations between changes in each morbidity burden z-scores and patient-related health outcomes from baseline to wave 3 (3–5 years later) in POPPY participants with HIV, assessed using linear or logistic regression models: Level 1 (unadjusted) and Level 2 (adjusted for age, sex, race, smoking, alcohol use and recreational drug use in the last 6 months).

	Patient-reported health outcomes			
	
Change in burden *z*-scores	Cognitive function (*n* = 709) *Beta* coefficient (95% CI) *P*-value	CES-D (*n* = 643) *Beta* coefficient (95% CI) *P*-value	PHQ-9 (*n* = 722) OR (95% CI) *P*-value	Pain (*n* = 656) OR (95% CI) *P*-value
*CVD*				
Level 1	−0.02 (−1.37, 1.33) *P* = 0.98	1.53 (−1.05, 4.12) *P* = 0.24	1.32 (0.92, 1.88) *P* = 0.13	1.42 (0.86, 2.36) *P* = 0.17
Level 2	−0.49 (−1.75, 0.77) *P* = 0.45	1.37 (−1.21, 3.95) *P* = 0.30	1.32 (0.92, 1.89) *P* = 0.13	1.34 (0.80, 2.25) *P* = 0.26
*Metabolic*				
Level 1	1.07 (−0.59, 2.72) *P* = 0.21	**3.37 (0.16, 6.59) *P* = 0.04**	1.23 (0.77, 1.96) *P* = 0.40	1.56 (0.80, 3.04) *P* = 0.20
Level 2	0.41 (−1.15, 1.98) *P* = 0.60	3.14 (−0.11, 6.38) *P* = 0.06	1.24 (0.77, 2.01) *P* = 0.37	1.43 (0.71, 2.86) *P* = 0.32
*Mental/Joint*				
Level 1	−0.33 (−1.69, 1.03) *P* = 0.63	**7.30 (4.81, 9.79) *P* *<* 0.001**	**1.99 (1.40, 2.82) *P* *<* 0.001**	1.53 (0.91, 2.59) *P* = 0.11
Level 2	−0.33 (−1.59, 0.94) *P* = 0.61	**7.08 (4.60, 9.57) *P* *<* 0.001**	**1.93 (1.35, 2.76) *P* *<* 0.001**	1.54 (0.90, 2.63) *P* = 0.11
*Neurological*				
Level 1	−0.35 (−1.31, 0.60) *P* = 0.47	**4.26 (2.36, 6.16) *P* *<* 0.001**	**1.48 (1.12, 1.95) *P* = 0.01**	**2.09 (1.47, 2.96) *P* *<* 0.001**
Level 2	−0.36 (−1.25, 0.53) *P* = 0.42	**4.35 (2.47, 6.24) *P* *<* 0.001**	**1.54 (1.16, 2.03) *P* *<* 0.001**	**2.08 (1.46, 2.98) *P* *<* 0.001**
*Cancer/Other*				
Level 1	0.15 (−0.86, 1.16) *P* = 0.77	**2.91 (0.90, 4.92) *P* *<* 0.001**	1.25 (0.95, 1.65) *P* = 0.11	**2.08 (1.45, 2.99) *P* *<* 0.001**
Level 2	−0.14 (−1.08, 0.80) *P* = 0.77	**3.28 (1.28, 5.27) *P* *<* 0.001**	**1.41 (1.06, 1.87) *P* = 0.02**	**2.20 (1.52, 3.19) *P* *<* 0.001**

CES-D, Center for Epidemiologic Studies Depression Scale; CI, confidence interval; CVDs, cardiovascular diseases; OR, odds ratio; PHQ-9, Patient Health Questionnaire-9. A 1-unit increase in morbidity z-scores represents an increase in burden by 1 standard deviation above the baseline mean. Bold text indicates statistically significant associations (*P* < 0.05). A grayscale shading system is used to reflect the relative strength of associations: darker cells represent stronger effect sizes (higher beta coefficients or odds ratios), and lighter cells indicate weaker but still statistically significant associations. Unshaded cells represent nonsignificant associations. This visual distinction is intended to aid interpretability and highlight key findings.

**Table 5:** Longitudinal associations between changes in each morbidity burden z-scores and healthcare utilization outcomes from baseline to wave 3 (3–5 years later) in POPPY participants with HIV, assessed using linear or logistic regression models: Level 1 (unadjusted) and Level 2 (adjusted for age, sex, race, smoking, alcohol use and recreational drug use in the last 6 months).

	Healthcare utilization outcomes (*n* = 793)			
	
Change in burden *z*-scores	GP visits IRR (95% CI) *P*-value	Specialist visits IRR (95% CI) *P*-value	ED visits IRR (95% CI) *P*-value	Hospital visits OR (95% CI) *P*-value
*CVD*				
Level 1	**1.21 (1.02, 1.44) *P* = 0.03**	**1.45 (1.10, 1.92) *P* = 0.01**	**1.45 (1.08, 1.94) *P* = 0.01**	1.58 (0.98, 2.54) *P* = 0.06
Level 2	1.18 (0.99, 1.41) *P* = 0.06	**1.47 (1.11, 1.94) *P* = 0.01**	**1.44 (1.06, 1.94) *P* = 0.02**	1.39 (0.86, 2.26) *P* = 0.18
*Metabolic*				
Level 1	**1.37 (1.10, 1.69) *P* *<* 0.001**	**1.74 (1.22, 2.48) *P* *<* 0.001**	**1.89 (1.29, 2.79) *P* *<* 0.001**	**3.47 (1.79, 6.74) *P* < 0.001**
Level 2	**1.35 (1.08, 1.67) *P* = 0.01**	**1.53 (1.07, 2.18) *P* = 0.02**	**1.89 (1.27, 2.81) *P* *<* 0.001**	**2.75 (1.41, 5.26) *P* = 0.003**
*Mental/joint*				
Level 1	**1.65 (1.41, 1.93) *P* *<* 0.001**	**1.50 (1.15, 1.95) *P* *<* 0.001**	1.30 (0.94, 1.78) *P* = 0.11	**3.82 (2.17, 6.72) *P* < 0.001**
Level 2	**1.66 (1.42, 1.95) *P* *<* 0.001**	**1.58 (1.22, 2.06) *P* *<* 0.001**	1.31 (0.95, 1.81) *P* = 0.09	**4.09 (2.30, 7.26) *P* < 0.001**
*Neurological*				
Level 1	**1.31 (1.16, 1.48) *P* *<* 0.001**	**1.62 (1.32, 1.99) *P* *<* 0.001**	**1.48 (1.19, 1.84) *P* *<* 0.001**	**2.17 (1.46, 3.24) *P* < 0.001**
Level 2	**1.3 (1.15, 1.47) *P* *<* 0.001**	**1.61 (1.32, 1.97) *P* *<* 0.001**	**1.47 (1.18, 1.84) *P* *<* 0.001**	**2.20 (1.46, 3.31) *P* < 0.001**
*Cancer/other*				
Level 1	**1.20 (1.05, 1.37) *P* = 0.01**	**1.62 (1.31, 2.00) *P* *<* 0.001**	1.27 (0.99, 1.62) *P* = 0.06	**2.38 (1.52, 3.72) *P* < 0.001**
Level 2	**1.22 (1.07, 1.38) *P* *<* 0.001**	**1.55 (1.26, 1.92) *P* *<* 0.001**	1.26 (0.98, 1.61) *P* = 0.07	**2.31 (1.47, 3.63) *P* < 0.001**

CI, confidence interval; CVDs, cardiovascular diseases; ED, emergency department; GP, general practitioner; IRR, incidence rate ratio; OR, odds ratio. A 1-unit increase in morbidity *z*-scores represents an increase in burden by 1 standard deviation above the baseline mean. Bold text indicates statistically significant associations (*P* < 0.05). A grayscale shading system is used to reflect the relative strength of associations: darker cells represent stronger effect sizes (higher beta coefficients or odds ratios), and lighter cells indicate weaker but still statistically significant associations. Unshaded cells represent nonsignificant associations. This visual distinction is intended to aid interpretability and highlight key findings.

The associations observed remained consistent when adjusting for HIV-related factors, including nadir CD4^+^ T-cell count and years since HIV diagnosis, with no significant changes in the estimates or direction of the associations (Supplementary Tables 3 and 4, Supplemental Digital Content).

## Discussion

This study underscores the significant and multifaceted impact of multimorbidity patterns on a broad range of health outcomes among people with HIV over a 3–5-year follow-up period. Distinct patterns demonstrated strong associations with physical and mental health, frailty and recurrent falls, and healthcare needs. These findings highlight the complex interplay between co-occurring morbidities and health trajectories, as well as the interconnected nature of physical and mental health challenges faced by this population over time.

*Mental/joint* and *neurological* patterns exhibited a strong association with frailty and recurrent falls, possibly due to their combined effects on physical, cognitive, and emotional functioning. Mental health conditions such as depression and anxiety can contribute to reduced physical activity and poor coordination [24,25], exacerbating frailty. Similarly, joint conditions like arthritis can impair mobility and balance, increasing fall risk [26]. Neurological disorders, including dizziness/vertigo, can directly impair spatial orientation and stability [27]. The synergistic effects of co-occurring morbidities can result in a cumulative burden, thereby increasing vulnerability and the risk of frailty and falls over time. These associations suggest that individuals with co-morbidities from these patterns could benefit from early frailty screening and fall-prevention interventions, including physiotherapy and support for balance and mobility. Interestingly, the *mental/joint* pattern was the only pattern not associated with functional impairment, suggesting that while mental health and joint conditions may significantly influence perceptions of overall health and physical well being overtime, they may not limit specific instrumental activities of daily living, assessed by the IADL scale. This difference may reflect the broader impact of pain, fatigue and psychosocial factors rather than direct physical disability.

Chronic inflammation and persistent pain associated with morbidities linked to the *mental/joint* pattern, such as joint inflammation/arthritis and bowel disorders [28–31], and the *neurological* pattern (e.g. encephalitis [32,33]), can exacerbate mental health problems [34–38]. This may explain the strong associations observed between both patterns and SF-36 mental health scores, as well as underscoring the complex interactions between physical and mental morbidities; as physical health worsens, this can heighten psychological distress and impact mental well being, which, in turn, can lead to a detrimental cycle. The interconnectedness and potentially bidirectional relationship between physical and mental health conditions were also highlighted by consistent associations between *mental/joint, neurological* and *cancer/other* scores with poorer depressive scores, by both CES-D and PHQ-9 questionnaires. These findings highlight the need for integrated mental health services, particularly for those with joint disorders, bowel disease, or neurological conditions. Although associations between *CVDs* and *metabolic* burden and depressive scores did not reach statistical significance, similar trends were observed.

The strong association of the *neurological* and *cancer/other* patterns with widespread pain underscores the persistent nature of pain across frequently co-occurring morbidities. Disorders within the *neurological* pattern, including encephalitis and psychosis, may contribute to widespread pain through various mechanisms, including inflammation and neurological dysfunction [39–41], and through secondary symptoms such as headaches and muscle tension (as suggested by the strong correlation with migraines/headaches). Similarly, the *cancer/other* pattern encompasses various malignancies and their associated treatments, which may induce widespread pain. Treatment-related side effects such as chemotherapy-induced neuropathy or radiation-induced tissue damage can contribute to pain in individuals with cancer [42–45]. Existing literature suggests that mental health problems, arthritis and inflammatory bowel disorders can also exacerbate pain perception/sensitivity and cause widespread pain and discomfort [46–50]. However, a significant association was not observed between higher *mental/joint* scores and widespread pain. This may be partly explained by relatively stable pain levels and/or established pain management strategies amongst individuals with chronic pain-related conditions, such as arthritis. Therefore, the lack of association over time may not necessarily indicate an absence of pain burden. Additionally, factors such as subjective interpretation of pain scales and recall bias may have also contributed to the lack of association.

Distinct healthcare utilization needs were also observed across the five multimorbidity patterns, each highlighting unique demands on the healthcare system. For instance, increased *CVD* burden was associated with more specialist and ED visits, reflecting the need for continuous cardiovascular monitoring (e.g. screening for CVD risk factors such as hypertension, dyslipidaemia and diabetes) and intervention. Similarly, an increase in *metabolic* burden was associated with all four healthcare utilization outcomes, underscoring the extensive ongoing management required for metabolic conditions such as diabetes and dyslipidaemia. These findings suggest a high frequency of interactions with multiple healthcare providers (e.g. specialists and allied professionals), reinforcing the need for coordinated care models. *mental/joint* and *neurological* patterns were associated with increased GP and hospital specialist visits, again suggesting the necessity of multidisciplinary input. Furthermore, an increase in *cancer/other* burden was associated with an increased number of specialist visits and higher odds of hospital visits. This reflects the complex and intensive nature of cancer care, often involving multidisciplinary teams and specialized treatments, resulting in frequent specialist consultations and hospital admissions for care.

### Strengths and limitations

This study is among the first to evaluate multimorbidity patterns in people with HIV across a comprehensive range of outcomes over 3–5-years. Beyond identifying associations, our findings demonstrate how specific multimorbidity patterns, such as *CVD, mental/joint* and *neurological* patterns, can be used to anticipate risks like frailty, depressive symptoms, and high healthcare utilization. These results have practical implications for clinical care, supporting the development of targeted, pattern-based interventions (e.g. fall prevention, mental health support, metabolic screening) and informing prognostic tools to stratify risk and guide personalized, multidisciplinary care in this ageing HIV population.

However, certain limitations must be considered. First, self-reported questionnaire data may be affected by social desirability bias, memory recall or misinterpretation of questionnaire items, particularly for sensitive topics like mental health, where stigma or personal perceptions may influence reporting. Second, the associations between *mental/joint* burden and depressive scores should be interpreted cautiously, as depression and anxiety (strongly correlated with the *mental/joint* pattern) can be detected through the CES-D and PHQ-9 questionnaires, which may overlap with self-reported medical history. Nevertheless, these associations could serve as a ‘control’, as one would expect individuals with higher *mental/joint* burden to report higher depressive scores. Third, the physical and mental health scores were standardized using age- and sex-specific norms from the 1996 Health Survey for England. While this allows comparability with existing studies, the lack of more recent population norms may limit relevance to current health levels. Fourth, although many associations were assessed, no adjustment for multiple comparisons was made due to the exploratory nature of the study. Methods like Bonferroni adjustment could obscure important findings, so results were interpreted cautiously, emphasizing consistency across patterns and outcomes and effect size rather than solely *P*-values. Finally, some outcomes may act as mediators. For example, widespread pain might influence the association between multimorbidity burden and healthcare utilization. Thus, further research is needed to explore these potential pathways.

## Conclusion

Our findings underscore the need for integrated, person-centred care models tailored to the specific multimorbidity patterns seen in ageing people with HIV. Rather than a one-size-fits-all approach, our results support the value of targeted, pattern-specific care strategies, such as fall-prevention programs for those with neurological burden and mental health conditions and proactive cardiovascular risk monitoring for those with CVD or metabolic burden. Aligning care delivery with these patterns may help healthcare systems better anticipate service demands, reduce fragmentation, and improve both clinical outcomes and quality of life for people living with HIV. Future research should explore how such pattern-informed care pathways can be implemented in routine practice.

## Acknowledgements

POPPY Management Group: Saumitro Deb, Nicki Doyle, Paddy Mallon, Maxine Ng, Claire Petersen, Frank Post, Caroline Sabin, Memory Sachikonye, Alan Winston POPPY Study Steering Committee: Jasmini Alagaratnam, Jane Anderson, David Asboe, Marta Boffito, Fiona Burns, Aoife Cotter, Saumitro Deb, Nicki Doyle, Eoin Feeney, Paddy Mallon, Carlos Mejia, Maxine Ng, Claire Petersen, Frank Post, Caroline Sabin, Memory Sachikonye, Jaime Vera, Laura Waters, Alan Winston POPPY Sites: USt Stephen's Centre, Chelsea and Westminster Hospital (Marta Boffito, Jasmini Alagaratnam, Ruth Byrne, Alexia Cheneau, Rosa Diaz Echeverria, Serge Fedele, Mohammed Hassan, Darwin Matila, Roya Movahedi, Beatrice Ouma, Javier Pinedo, Kathleen Ridor, Jad Salha, Sharom Scarpetta, Reda Yassein) Homerton Sexual Health Services, Homerton University Hospital (Jane Anderson, Tracey Fong, Lidia Ignatov, Jagrul Miah, Iain Reeves) Caldecot Centre, King's College Hospital (Frank Post, Birgit Barbini, Lucy Campbell, Luella Hanbury, Rachel Hung, Leigh McQueen, Zoe Ottaway, Daniel Trotman, Emily Wandolo) Mortimer Market Centre, Central and Northwest London NHS Foundation Trust (Laura Waters, Fowsiya Nur, Brittney Prusty, Sandra Coombes, Gosala Gopalakrishnan, Ebunoluwa Taiwo, Christos Karathanasis, Laura Hennelly, Claudia Benatar, Alejandro Arenas Pinto) Ian Charleson Day Centre, Royal Free Hospital (Fiona Burns, Tom Allan, Jonathan Edwards, Tom Fernandez, Jia Bo He, Katie Spears, Abigail Tobin) Clinical Research Facility, Royal Sussex County Hospital (Jaime Vera, Tanya Adams, Lisa Barbour, Kay Franklin, Chioma Iwuchukwu, Donards Tanedo, Andrea Terlingo, Vittorio Trevitt, Mikaela Vinick) Clinical Trials Centre, St. Mary's Hospital London (Alan Winston, Wilbert Ayap, Merle Henderson, Ishrat Jahan, Ian McGuinness, Idah Mojela, Marcelino Molina, Jacquie Ujetz) Catherine McAuley Centre, Mater Misericordiae University Hospital (Carlos Mejia Nijat Ahmadi, Alejandro Garcia, Binsa Jose) St Vincent's University Hospital (Eoin Feeney, Alejandro Garcia, Fiorina Rigonat, Stefano Savinelli) POPPY methodology/statistics: Caroline Sabin, Saumitro Deb, Hajra Okhai, Luxsena Sukumaran. We acknowledge the use of the National Institute for Health Research (NIHR)/Wellcome Trust Clinical Research Facility at King's College Hospital. The research is also funded by the National Institute for Health Research Biomedical Research Centre based at Imperial College Healthcare NHS Trust and Imperial College London. Supported in part by research grants from Investigator-Initiated Studies Program of Merck Sharp & Dohme LLC, ViiV Healthcare and Gilead Sciences. NIHR HPRU Steering Committee: Professor CA Sabin (HPRU Director), Dr J Saunders (UKHSA Lead), Professor C Mercer, Dr H Mohammed, Professor G Rait, Dr R Simmons, Professor W Rosenberg, Dr T Mbisa, Professor R Raine, Dr S Mandal, Dr R Yu, Dr S Ijaz, Dr F Lorencatto, Dr R Hunter, Dr K Foster and Dr M Tahir.

Ethical considerations: The study was approved by the UK National Research Ethics Service (NRES; Fulham, London, UK, number 12/ LO/1409).

Consent to participate: All participants provided written informed consent.

Funding statement: The POPPY study waves 1 to 3 was funded by investigator-initiated grants from BMS, Gilead Sciences, Janssen, Merck and ViiV Healthcare (EudraCT Number: 2012-003581–40; Sponsor Protocol Number: CRO1992). The POPPY study waves 4 and 5 is funded by investigator-initiated grants from Gilead Sciences, ViiV Healthcare and Merck Sharp & Dohme (UK) Limited (Sponsor protocol number: 19SM5112). We acknowledge the use of the National Institute for Health Research (NIHR)/Wellcome Trust Clinical Research Facility at King's College Hospital. The study is also supported by the National Institute for Health Research (NIHR) Biomedical Research Centre based at Imperial College Healthcare NHS Trust and Imperial College London and by an NIHR Senior Investigator Award to Professor C. A. Sabin. LS was funded through the National Institute for Health and Care Research Health Protection Research Unit (NIHR HPRU) in Blood Borne and Sexually Transmitted Infections at University College London in partnership with the UK Health Security Agency (HPRU Grant no: NIHR200911). The views expressed are those of the author(s) and not necessarily those of the NHS, the NIHR, the Department of Health or the funders.

### Data availability

The authors confirm that the data supporting the findings of this study are available within the article and/or its supplementary materials.

### Conflicts of interest

C.S. has received funding from Gilead Sciences, ViiV Healthcare, MSD and Janssen-Cilag for membership of Advisory Boards and for preparation of educational materials. A.W. has been an investigator on studies sponsored by, received research grants from and received speaker fees or honoraria from ViiV Healthcare, Janssen, Gilead Sciences and MSD. F.A.P. reports grants and personal fees from Gilead Sciences, ViiV Healthcare and MS; all outside of the work reported here. P.W.G.M. has received honoraria and/or travel grants from Gilead Sciences, MSD, Bristol-Myers Squibb, and ViiV Healthcare, and has been awarded grants by Science Foundation Ireland, outside the submitted work. J.A. reports personal fees from Gilead Sciences and ViiV; all outside of the work reported here. M.B. has acted as a speaker or adviser to, has been an investigator for, or has received grants to her institution from Gilead, ViiV, Janssen, B.M.S., Teva, Cipla, Mylan, and MSD; all outside the work presented here. J. V. reports travel, research grants, and personal fees from Merck, Janssen Cilag, Piramal Imaging, ViiV Healthcare, and Gilead; all outside the work presented here. L.W. has received speaker or advisory fees from ViiV, Merck & Janssen. She is an investigator on trials sponsored by Gilead, ViiV & Merck. F.B. has received and institutional grant and speakers fees from Gilead Sciences Ltd; both outside the work presented here. The funders were not involved in the study design, collection, analysis, interpretation of data, the writing of this article or the decision to submit it for publication. All authors declare no other competing interests.

## Supplementary Material

Supplemental Digital Content
