# Supplementary material for: Changes in multimorbidity burden and their impact on patient and healthcare outcomes in people with HIV over a 3–5-year period
Source: AIDS. 2025 Jun 4;39(12):1784–93. doi: 10.1097/QAD.0000000000004260 (PMC12404628; doi:10.1097/QAD.0000000000004260)
Supplement: Supplemental Digital Content [file aids-39-1784-s001.docx]

**SUPPLEMENTARY MATERIAL**

**Supplementary Table 1.** List of 70 comorbidities (from 19 organ system/pathogenic groups) considered in the main analysis, with a prevalence >1.5% in all POPPY participants with HIV (n=1,073) at baseline

| **Organ system** | **Comorbidities** | **N (%)** |
| --- | --- | --- |
| AIDS events | AIDS-related cancers | 19 (1.8) |
|  | CMV | 28 (2.6) |
|  | Kaposi's sarcoma | 70 (6.5) |
|  | Other AIDS events | 113 (10.5) |
|  | PCP | 96 (8.9) |
|  | Tuberculosis | 78 (7.3) |
| Haematological | Anaemia | 25 (2.3) |
|  | DVT | 22 (2.1) |
| Respiratory | Asthma/bronchitis/COPD | 263 (24.5) |
|  | Hay fever | 85 (7.9) |
|  | Chest infections | 115 (10.7) |
|  | Pneumonia | 53 (4.9) |
| Cancer | Haematological cancer | 13 (1.2) |
|  | Skin cancer | 45 (4.2) |
|  | Solid organ cancer | 55 (5.1) |
| Cardiovascular | Arrhythmia | 38 (3.5) |
|  | CABG | 24 (2.2) |
|  | Dyslipidemia | 304 (28.3) |
|  | Heart failure | 23 (2.1) |
|  | Hypertension | 245 (22.8) |
|  | IHD | 46 (4.3) |
|  | Myocardial infarction | 37 (3.4) |
|  | Peripheral vascular disease | 20 (1.9) |
|  | CVA/TIA | 37 (3.4) |
| Mental health | Anxiety/Panic attacks | 96 (8.9) |
|  | Clinical depression | 346 (32.2) |
|  | Depressive symptoms | 84 (7.8) |
|  | Psychosis | 18 (1.7) |
|  | Sleeping problems | 76 (7.1) |
| Ear problems | Ear dysfunction | 61 (5.7) |
| Endocrine | Type II diabetes | 56 (5.2) |
|  | Erectile dysfunction | 78 (7.3) |
|  | Hypogonadism | 42 (3.9) |
|  | Lipodystrophy | 27 (2.5) |
|  | Hypothyroidism | 26 (2.4) |
| Eye problems | Eye problems | 88 (8.2) |
| Gastrointestinal | Persistent bowel disorders | 233 (21.7) |
|  | Hepatitis A | 45 (4.2) |
|  | Hepatitis B | 199 (18.5) |
|  | Hepatitis C | 101 (9.4) |
|  | Hernia | 42 (3.9) |
|  | Liver problems | 76 (7.1) |
|  | GORD | 82 (7.6) |
| Genitourinary | Urinary incontinence | 43 (4) |
|  | Kidney stones | 31 (2.9) |
|  | Prostate dysfunction | 37 (3.4) |
|  | Renal problems | 54 (5) |
|  | UTI | 34 (3.2) |
| Infections | Fungal infections | 52 (4.8) |
|  | HSV/VZV | 90 (8.4) |
| Joint and bone | Aches and pains | 131 (12.2) |
|  | Joint inflammation/Arthritis | 192 (17.9) |
|  | Joint replacement | 25 (2.3) |
|  | Osteopenia/osteoporosis | 85 (7.9) |
| Neurological | Encephalitis | 17 (1.6) |
|  | Epilepsy | 43 (4) |
|  | Loss consciousness | 31 (2.9) |
|  | Migraine/headaches | 52 (4.8) |
|  | Peripheral neuropathy | 238 (22.2) |
|  | Dizziness/vertigo | 116 (10.8) |
| Skin | Eczema | 115 (10.7) |
|  | Pruritus | 21 (2) |
|  | Psoriasis | 49 (4.6) |
| STIs | Chlamydia | 296 (27.6) |
|  | Gonorrhoea | 458 (42.7) |
|  | HPV | 105 (9.8) |
|  | HSV | 155 (14.4) |
|  | LGV | 45 (4.2) |
|  | Syphilis | 327 (30.5) |
| Vitamin deficiency | Vitamin D deficiency | 24 (2.2) |
| *Abbreviations:* ***AIDS****, Acquired immunodeficiency syndrome;* ***CMV****, Cytomegalovirus;* ***PCP****, Pneumocystis pneumonia;* ***DVT****, Deep vein thrombosis;* ***COPD****, Chronic obstructive pulmonary disease;* ***CABG****, Coronary artery bypass grafting;* ***IHD****, Ischemic heart disease;* ***CVA/TIA****, Cerebrovascular accident/transient ischaemic attack;* ***GORD****, Gastro-oesophageal reflux disease;* ***UTI****, Urinary tract infection;* ***HSV/VZV****, Herpes simplex virus/varicella zoster virus;* ***HPV****, Human papillomavirus;* ***LGV****, Lymphogranuloma venereum* | | |

**Supplementary Table 2.** Morbidity patterns identified using principal component analysis (PCA) in all POPPY participants with HIV (n=1,073)

| PC (% of variance explained) | Label | Comorbidities correlated with PC |
| --- | --- | --- |
| 1 (6.5%) | CVDs | CABG/PCTA (0.68), Heart failure (0.62), Hypertension (0.70), IHD (0.72), Myocardial infarction (0.69), Peripheral vascular disease (0.46), Renal problems (0.40) |
| 2 (4.5%) | STIs | Gonorrhoea (0.78), Chlamydia (0.68), LGV (0.64), Syphilis (0.64), HSV (0.48) |
| 3 (3.8%) | Metabolic | Peripheral neuropathy (0.57), Type 2 diabetes (0.57), Hypothyroidism (0.46), Dyslipidaemia (0.45), Pruritis (0.41) |
| 4 (3.1%) | Mental/Joint | Clinical depression (0.75), Anxiety/Panic attacks (0.50), Joint inflammation/ Arthritis (0.45), Joint replacement (0.45), Sleeping problems (0.40), Bowel disorders (0.40) |
| 5 (2.9%) | Neurological | Dizziness/Vertigo (0.61), Encephalitis (0.60), Loss of consciousness (0.40) |
| 6 (2.6%) | Cancer/Other | Haematological cancer (0.64), Hernia (0.45), Osteopenia/osteoporosis (0.44), AIDS-related cancer (0.43) |
| *Abbreviations: PC, principal component; CABG/PCTA, Coronary artery bypass grafting/percutaneous transluminal coronary angioplasty; IHD, Ischemic heart disease; LGV, Lymphogranuloma venereum; HSV, Herpes simplex virus; AIDS, Acquired immunodeficiency syndrome*  *Note: Values in parentheses represent factor loadings from the PCA, indicating the strength and direction of correlation between each comorbidity and the corresponding pattern (PC).* | | |

**Supplementary Table 3a.** Longitudinal associations between changes in each morbidity burden z-scores and patient-related health outcomes from baseline to wave 3 (3-5 years later) in POPPY participants with HIV, assessed using linear or logistic regression models: Level 1 (adjusted for age, sex, race, smoking, alcohol use and recreational drug use in the last six months) and Level 2 (adjusted for factors in level 1 plus nadir CD4+ T-cell count and years since HIV diagnosis)

|  | **Patient-related health outcomes** | | | | |
| --- | --- | --- | --- | --- | --- |
| Change in burden z-scores | **Physical health (*n***=**618)**  *Beta* coefficient (95% CI)  *p*-value | **Mental health (*n***=**618)**  *Beta* coefficient (95% CI)  *p*-value | **Frailty (*n***=**793)**  OR (95% CI) *p*-value | **Recurrent falls (*n***=**714)**  OR (95% CI) *p*-value | **Functional impairment (*n***=**758)**  OR (95% CI) *p*-value |
| ***CVDs*** |  |  |  |  |  |
| Level 1 | -2.71 (-4.89, -0.52) *p*=0.02 | -1.38 (-3.56, 0.79) *p*=0.21 | 1.16 (0.75, 1.78) *p*=0.51 | 1.47 (0.91, 2.39) *p*=0.12 | 1.96 (1.22, 3.18) *p*=0.01 |
| Level 2 | -2.46 (-4.62, -0.31) *p*=0.03 | -1.19 (-3.35, 0.97) *p*=0.28 | 1.13 (0.73, 1.75) *p*=0.58 | 1.45 (0.88, 2.37) *p*=0.14 | 1.87 (1.15, 3.05) *p*=0.01 |
| ***Metabolic*** |  |  |  |  |  |
| Level 1 | -1.59 (-4.37, 1.20) *p*=0.26 | -3.11 (-5.87, -0.36) *p*=0.03 | 1.69 (0.99, 2.89) *p*=0.05 | 0.87 (0.44, 1.75) *p*=0.71 | 2.64 (1.45, 4.81) *p<*0.001 |
| Level 2 | -1.06 (-3.81, 1.69) *p*=0.45 | -2.73 (-5.47, 0.02) *p*=0.05 | 1.66 (0.97, 2.83) *p*=0.06 | 0.83 (0.41, 1.67) *p*=0.6 | 2.53 (1.38, 4.61) *p<*0.001 |
| ***Mental/Joint*** |  |  |  |  |  |
| Level 1 | -3.91 (-6.05, -1.78) *p<*0.001 | -7.13 (-9.20, -5.07) *p<*0.001 | 1.93 (1.27, 2.91) *p<*0.001 | 1.72 (1.09, 2.74) *p*=0.02 | 1.69 (1.00, 2.85) *p*=0.05 |
| Level 2 | -3.42 (-5.54, -1.31) *p<*0.001 | -6.81 (-8.87, -4.75) *p<*0.001 | 1.89 (1.25, 2.87) *p<*0.001 | 1.63 (1.02, 2.61) *p*=0.04 | 1.59 (0.94, 2.69) *p*=0.09 |
| ***Neurological*** |  |  |  |  |  |
| Level 1 | -4.55 (-6.13, -2.98) *p<*0.001 | -3.01 (-4.60, -1.43) *p<*0.001 | 1.88 (1.39, 2.54) *p<*0.001 | 1.49 (1.06, 2.10) *p*=0.02 | 2.10 (1.50, 2.93) *p<*0.001 |
| Level 2 | -4.45 (-6.01, -2.90) *p<*0.001 | -2.93 (-4.50, -1.35) *p<*0.001 | 1.87 (1.38, 2.52) *p<*0.001 | 1.49 (1.06, 2.11) *p*=0.02 | 2.07 (1.48, 2.91) *p<*0.001 |
| ***Cancer/Other*** |  |  |  |  |  |
| Level 1 | -3.07 (-4.74, -1.40) *p<*0.001 | -2.41 (-4.07, -0.75) *p<*0.001 | 1.65 (1.20, 2.28) *p<*0.001 | 1.25 (0.86, 1.82) *p*=0.25 | 1.57 (1.07, 2.29) *p*=0.02 |
| Level 2 | -2.75 (-4.40, -1.10) *p<*0.001 | -2.17 (-3.82, -0.51) *p*=0.01 | 1.63 (1.18, 2.26) *p<*0.001 | 1.20 (0.82, 1.76) *p*=0.35 | 1.49 (1.01, 2.19) *p*=0.04 |
| Abbreviations: **OR**, odds ratio; **CI**, confidence interval; **CVDs**, cardiovascular diseases  Note: A 1-unit increase in morbidity z-scores represents an increase in burden by 1 standard deviation above the baseline mean | | | | | |

**Supplementary Table 3b.** Longitudinal associations between changes in each morbidity burden z-scores and patient-related health outcomes from baseline to wave 3 (3-5 years later) in POPPY participants with HIV, assessed using linear or logistic regression models: Level 1 (adjusted for age, sex, race, smoking, alcohol use and recreational drug use in the last six months) and Level 2 (adjusted for factors in level 1 plus nadir CD4+ T-cell count and years since HIV diagnosis)

|  | **Patient-reported health outcomes** | | | | |
| --- | --- | --- | --- | --- | --- |
| Change in burden z-scores | **Cognitive function (*n***=**709)**  *Beta* coefficient (95% CI)  *p*-value | **CES-D (*n***=**643)**  *Beta* coefficient (95% CI)  *p*-value | **PHQ-9 (*n***=**722)**  OR (95% CI) *p*-value | | **Pain (*n***=**656)**  OR (95% CI) *p*-value |
| ***CVDs*** |  |  | |  |  |
| Level 1 | -0.49 (-1.75, 0.77) *p*=0.45 | 1.37 (-1.21, 3.95) *p*=0.30 | | 1.32 (0.92, 1.89) *p*=0.13 | 1.34 (0.80, 2.25) *p*=0.26 |
| Level 2 | -0.58 (-1.85, 0.69) *p*=0.37 | 1.02 (-1.54, 3.58) *p*=0.43 | | 1.27 (0.88, 1.83) *p*=0.19 | 1.30 (0.77, 2.21) *p*=0.33 |
| ***Metabolic*** |  |  | |  |  |
| Level 1 | 0.41 (-1.15, 1.98) *p*=0.60 | 3.14 (-0.11, 6.38) *p*=0.06 | | 1.24 (0.77, 2.01) *p*=0.37 | 1.43 (0.71, 2.86) *p*=0.32 |
| Level 2 | 0.40 (-1.17,1.96) *p*=0.62 | 2.58 (-0.64, 5.79) *p*=0.12 | | 1.12 (0.69, 1.81) *p*=0.65 | 1.35 (0.66, 2.75) *p*=0.41 |
| ***Mental/Joint*** |  |  | |  |  |
| Level 1 | -0.33 (-1.59, 0.94) *p*=0.61 | 7.08 (4.60, 9.57) *p<*0.001 | | 1.93 (1.35, 2.76) *p<*0.001 | 1.54 (0.90, 2.63) *p*=0.11 |
| Level 2 | -0.37 (-1.65, 0.90) *p*=0.57 | 6.56 (4.08, 9.04) *p<*0.001 | | 1.77 (1.23, 2.54) *p<*0.001 | 1.44 (0.83, 2.51) *p*=0.20 |
| ***Neurological*** |  |  | |  |  |
| Level 1 | -0.36 (-1.25, 0.53) *p*=0.42 | 4.35 (2.47, 6.24) *p<*0.001 | | 1.54 (1.16, 2.03) *p<*0.001 | 2.08 (1.46, 2.98) *p<*0.001 |
| Level 2 | -0.40 (-1.29, 0.49) *p*=0.38 | 4.14 (2.26, 6.01) *p<*0.001 | | 1.52 (1.15, 2.02) *p<*0.001 | 2.21 (1.52, 3.20) *p<*0.001 |
| ***Cancer/Other*** |  |  | |  |  |
| Level 1 | -0.14 (-1.08, 0.80) *p*=0.77 | 3.28 (1.28, 5.27) *p<*0.001 | | 1.41 (1.06, 1.87) *p*=0.02 | 2.20 (1.52, 3.19) *p<*0.001 |
| Level 2 | -0.16 (-1.11, 0.78) *p*=0.73 | 2.87 (0.88, 4.86) *p<*0.001 | | 1.35 (1.01, 1.80) *p*=0.04 | 2.14 (1.45, 3.14) *p<*0.001 |
| Abbreviations:  **CES-D**, Center for Epidemiologic Studies Depression Scale; **PHQ-9**, Patient Health Questionnaire-9; **OR**, odds ratio; **CI**, confidence interval; **CVDs**, cardiovascular diseases  Note: A 1-unit increase in morbidity z-scores represents an increase in burden by 1 standard deviation above the baseline mean | | | | | |

**Supplementary Table 4.** Longitudinal associations between changes in each morbidity burden z-scores and healthcare utilisation outcomes from baseline to wave 3 (3-5 years later) in POPPY participants with HIV, assessed using linear or logistic regression models: Level 1 (adjusted for age, sex, race, smoking, alcohol use and recreational drug use in the last six months) and Level 2 (adjusted for factors in level 1 plus nadir CD4+ T-cell count and years since HIV diagnosis)

|  | **Healthcare utilisation outcomes (*n***=**794)** | | | | |
| --- | --- | --- | --- | --- | --- |
| Change in burden z-scores | **GP visits**  IRR (95% CI) *p*-value | **Specialist visits**  IRR (95% CI) *p*-value | **ED visits**  IRR (95% CI) *p*-value | | **Hospital visits**  OR (95% CI) *p*-value |
| ***CVDs*** |  |  | |  |  |
| Level 1 | 1.18 (0.99, 1.41) *p*=0.06 | 1.47 (1.11, 1.94) *p*=0.01 | | 1.44 (1.06, 1.94) *p*=0.02 | 1.58 (0.98, 2.54) p=0.06 |
| Level 2 | 1.14 (0.96, 1.36) *p*=0.13 | 1.47 (1.11, 1.95) *p*=0.01 | | 1.37 (1.02, 1.85) *p*=0.04 | 1.30 (0.79, 2.13) p=0.30 |
| ***Metabolic*** |  |  | |  |  |
| Level 1 | 1.35 (1.08, 1.67) *p*=0.01 | 1.53 (1.07, 2.18) *p*=0.02 | | 1.89 (1.27, 2.81) *p<*0.001 | 3.47 (1.79, 6.74) p<0.001 |
| Level 2 | 1.31 (1.06, 1.63) *p*=0.01 | 1.48 (1.04, 2.12) *p*=0.03 | | 1.82 (1.23, 2.69) *p<*0.001 | 2.73 (1.38, 5.39) p=0.004 |
| ***Mental/Joint*** |  |  | |  |  |
| Level 1 | 1.66 (1.42, 1.95) *p<*0.001 | 1.58 (1.22, 2.06) *p<*0.001 | | 1.31 (0.95, 1.81) *p*=0.09 | 3.82 (2.17, 6.72) p<0.001 |
| Level 2 | 1.63 (1.39, 1.90) *p<*0.001 | 1.56 (1.20, 2.02) *p<*0.001 | | 1.28 (0.93, 1.76) *p*=0.13 | 3.84 (2.14, 6.89) p<0.001 |
| ***Neurological*** |  |  | |  |  |
| Level 1 | 1.3 (1.15, 1.47) *p<*0.001 | 1.61 (1.32, 1.97) *p<*0.001 | | 1.47 (1.18, 1.84) *p<*0.001 | 2.17 (1.46, 3.24) p<0.001 |
| Level 2 | 1.29 (1.14, 1.45) *p<*0.001 | 1.60 (1.31, 1.96) *p<*0.001 | | 1.46 (1.17, 1.82) *p<*0.001 | 2.13 (1.41, 3.24) p<0.001 |
| ***Cancer/Other*** |  |  | |  |  |
| Level 1 | 1.22 (1.07, 1.38) *p<*0.001 | 1.55 (1.26, 1.92) *p<*0.001 | | 1.26 (0.98, 1.61) *p*=0.07 | 2.38 (1.52, 3.72) p<0.001 |
| Level 2 | 1.21 (1.06, 1.38) *p<*0.001 | 1.56 (1.26, 1.92) *p<*0.001 | | 1.22 (0.95, 1.56) *p*=0.12 | 2.24 (1.41, 3.58) p=0.001 |
| Abbreviations: **GP**, general practitioner; **ED**, Emergency department; **IRR**, Incidence Rate Ratio; **OR**, odds ratio; **CI**, confidence interval; **CVDs**, cardiovascular diseases  Note: A 1-unit increase in morbidity z-scores represents an increase in burden by 1 standard deviation above the baseline mean | | | | | |
